# Erratum: Correspondence: Enhancing a phase measurement by sequentially probing a solid-state system

**DOI:** 10.1038/ncomms11864

**Published:** 2016-06-07

**Authors:** P. A. Knott, W. J. Munro, J. A. Dunningham

Nature Communications 7: Article number: 11520 10.1038/ncomms11520 (2016); Published: 0592016; Updated: 06072016

The HTML version of this Correspondence was previously published with an incomplete title and incorrect Article number. This has now been corrected in the HTML; the PDF version of the paper was correct from the time of publication.

